# The Outcomes of a 12-Week Internet Intervention Aimed at Improving Fitness and Health-Related Quality of Life in Overweight Adolescents: The Young & Active Controlled Trial

**DOI:** 10.1371/journal.pone.0114732

**Published:** 2014-12-05

**Authors:** Kirsti Riiser, Knut Løndal, Yngvar Ommundsen, Milada Cvancarova Småstuen, Nina Misvær, Sølvi Helseth

**Affiliations:** 1 Norwegian School of Sport Sciences, Oslo, Norway; 2 Oslo and Akershus University College of Applied Sciences, Oslo, Norway; Weill Cornell Medical College Qatar, Qatar

## Abstract

**Background:**

Overweight and obesity among adolescents may have consequences, with potentially lasting effects on health and health-related quality of life (HRQoL). Excess weight is also associated with decreases in physical activity and cardiorespiratory fitness. The aim of the current study was to investigate the short-term effects of a 12-week Internet intervention in a primary care setting intended to increase cardiorespiratory fitness and HRQoL among overweight and obese adolescents.

**Methods:**

In this controlled trial, participants (13–15 years) were non-randomly allocated to an intervention- or a control group. The intervention group received 12-weeks access to an online program providing tailored physical activity counseling based on principles from Self-determination Theory and Motivational Interviewing. The control group received standard follow-up by the school nurses. The primary outcome measure of cardiorespiratory fitness was determined using a shuttle run test. The secondary outcomes: HRQoL, leisure time exercise, body image and self-determined motivation for physical activity and exercise, were assessed by self-report measures. Age- and gender-adjusted body mass index (BMI) was calculated based on measurements of height and weight. To compare pre-to post intervention differences within groups, a paired samples t-test was used while crude differences between groups were analyzed with an independent samples t-test.

**Results:**

Of the 120 participants, 108 completed the study, 75 in the intervention group and 33 in the control group. Exposure to the intervention had a small effect on cardiorespiratory fitness (0.14; 95% CI [0.01;0.28]; *P* = 0.04), and a moderate effect on HRQoL (5.22; 95% CI [0.90; 9.53]; *P* = 0.02). Moreover, the control group increased significantly in BMI, yielding a moderate preventive effect on BMI (−0.39; 95% CI [−0.74;−0.03]; *P* = 0.03) for the intervention group.

**Conclusion:**

The results suggest that the Internet intervention with tailored physical activity counseling can have beneficial short-term effect on cardiorespiratory fitness, HRQoL and BMI among adolescents with overweight and obesity.

**Trial Registration:**

ClinicalTrials.gov NCT01700309

## Background

Overweight and obesity in children and adolescents is seen as a significant public health problem worldwide [1]. Excess weight is not merely a physical health matter. Overweight and obesity may have considerable psychosocial consequences with potentially lasting effects on well-being [2], [3]. The stigma associated with being overweight can be pervasive and may mediate emotional and social problems [4]. Studies have documented that overweight and obesity in adolescence are related to lower self-esteem and depressive symptoms, which affect health-related quality of life (HRQoL) [3], [5], [6]. Being overweight have also been found to be associated with a decrease in physical activity (PA) and fitness indicating that being overweight might lead to inactivity just as much as inactivity leads to being overweight [7], [8]. PA is considered a key component in prevention and treatment of overweight and obesity. The adverse effects of fatness on cardiovascular risk are found to be counteracted by higher levels of cardiorespiratory fitness (CRF) among adolescents [9], [10]. Moreover, it is equally important that PA and fitness are associated with well-being and HRQoL [11]. So far, few studies have reported on associations between HRQoL and PA levels among overweight adolescents. However there are indications of such a relationship, showing that physically active overweight children demonstrate higher HRQoL compared to those who are less active [12]. CRF is also documented to be related to HRQoL among overweight and obese children and adolescents suggesting that improving fitness could be a strategy for increasing HRQoL [13], [14]. These findings underline the importance of developing and making available targeted interventions that promote PA and fitness and potentially contribute to increase HRQoL in addition to preventing further weight gain among overweight adolescents.

### Physical activity and web-based interventions

Although it seems that the field of primary preventive interventions is moving forward, and beneficial effects of programs on BMI have been found [15], very few secondary preventive studies targeting those already overweight have been conducted in primary care [16]. In the search for new effective and efficient strategies to change and maintain health behavior, web-based interventions are becoming increasingly popular. Web-delivered interventions give professionals the opportunity to offer interactive feedback to large numbers of people, while also making the messages relevant to each user by individualizing them [17]–[19]. Internet interventions for prevention and management of overweight and obesity in children and adolescents have shown promise in terms of either dietary and/or PA behavior [20], [21]. Reviews of internet-delivered interventions focusing exclusively on increasing PA among children and adolescents have also documented that such interventions could be feasible [22], [23]. However, the quality of studies of web-based PA and overweight interventions has been limited, and they have problems with achieving clinically meaningful long-term results [20]. In addition, the attrition rate is often reported to be high [18]. Given that there is a dose-response relationship between use and effectiveness, it is decisive to develop the interventions in ways that increase adherence [19]. In order to do so, it has been repeatedly stressed that interventions should consider individual tailoring, increased frequency of personal contact and extensive use of theoretical frameworks [17], [20], [22], [23].

### Theoretical basis

Self-determination Theory (SDT) has shown to be useful in understanding motivational, cognitive and affective processes of PA [24]. According to the theory, developing a sense of autonomy, competence and relatedness, is essential to make a person more self-regulated and able to sustain behavior [25]. Autonomy reflects the need to engage in activities with a sense of choice, competence represents the feeling that one can accomplish tasks and reach goals, while relatedness refers to the sense of being understood and respected by significant others [26]. Autonomy support, structure and interpersonal involvement can support the basic psychological needs of autonomy, competence and relatedness, and thus facilitate adoption and maintenance of physical activity [27]. SDT proposes that people can be intrinsically as well as extrinsically motivated in their regulation of behavior [25]. Different forms of motivation can be conceptualized along a continuum from non-autonomous controlled forms of behavior to completely autonomous forms [28]. Autonomous regulation of behavior is held to be more stable and enduring in addition to having more positive effects on human well-being than controlled regulation [25]. The social-environmental factors considered by SDT to facilitate self-determined function, such as autonomy support, have been shown to be closely related to the practice of motivational interviewing (MI) [29]. MI is a collaborative, person-centered form of guiding to elicit and strengthen motivation for change [30]. The MI-approach seeks to empower the participants' own reasons for change by expressing empathy, increasing awareness of discrepancies between goals and actions and supporting self-efficacy [29]. Development of the present intervention was informed by SDT and supplementary perspectives on self-regulation [31]. Inputs from SDT focusing on autonomy support, coupled with principles from MI, were used to promote behavior change in the tailored counseling (Table 1).

**Table 1:** Examples of autonomy supportive counselling and MI elements in the feedback.

Principles from autonomy supportive counseling:	Examples of principles from MI:	Examples of practical use:	Examples of feedback provided in Young & Active:
Support autonomy	Explore the adolescent's own reasons for change	Let the adolescent explore his/her own reasons for being physically active and exercising.	I am happy to read that you are satisfied with your training on Wednesday. You managed to do both running and weight exercises! Your goal is to complete what you plan for, and on Wednesday, you really did! Why? What made you do so? It is smart to think all this through; “What does it take for me to be as physically active as planned?”
Provide structure	Develop goals	Help set goals for PA and exercise. Make sure the goals are appropriate, realistic, and achievable.	To increase your aerobic fitness, which is one of your goals, you have to improve your lung capacity. This requires that you choose vigorous activities that increase your heart rate. Dancing is great exercise, but often contains frequent pausing, while cycling, running or swimming require that you keep your pace up over time. I challenge you to give it your best whenever you can, i.e. in the PE-class. Having said that, all the exercise and activity that you have planned, including dancing, is good for your fitness, with respect to strength, balance and flexibility.
Be involved	Express empathy	Display interest in the adolescent and his/her well-being.	I hope you are feeling better. I see from your registrations that some of your planned activities were cancelled because you were ill. It is important not to exert yourself too much when you have an infection. When you feel up to it, you can slowly increase the exercise intensity. Start carefully, do not overdo it.

## Aim

The aim of the current study was to investigate the short-term effects of a 12-week Internet intervention in a primary care setting intended to increase cardiorespiratory fitness and HRQoL among overweight and obese adolescents. We hypothesized that focusing on self-determined PA through an Internet intervention would have positive effects on the adolescents' cardiorespiratory fitness and HRQoL. Moreover, we hypothesized that compared to the control group, the intervention group would show increased self-determined motivation for PA and exercise, increased moderate to vigorous PA during leisure time, improved body image and stabilized or reduced BMI after the intervention.

## Methods

### Study design, recruitment and sample

The protocol for this trial and supporting TREND checklist are available as supporting information; see Protocol S1 and Checklist S1. This study was designed as a controlled trial without randomization. The initial randomization protocol was abandoned after responses from school nurses in the early stages of the study. They found it difficult to approach a sensitive topic like overweight by introducing the adolescents to an intervention they assigned for and hoped to receive, but perhaps ended up *not* receiving. Both their weight and their young age, make these adolescents especially vulnerable [32]. When designing the study as a control group study, the amount of information was reduced and adjusted specifically to the two different groups. In addition, the adolescents would immediately know for which group they were offered participation. Participants were allocated to intervention- and control groups sequentially. Recruitment of the intervention group was completed first, in February and March 2012 and between November 2012 and March 2013, and the control group between November and March the following school year. All participants filled in questionnaires three times: at baseline (T0), immediately after the intervention at 12 weeks (T1) and one year after baseline (T2). The current findings are based on the T0-T1 data. Adolescents identified as overweight or obese after standardized screening of weight and height in the eighth grade or follow-up measurements in the ninth grade [33], [34] and who were not engaged in other programs, were eligible for the study. We requested permission from primary health care head nurses of municipalities in three counties in eastern Norway to engage their school nurses in the recruitment of participants. Despite the fact that a great majority of the head nurses gave their permission, most school nurses declined our request, giving reasons such as lack of resources, no priority given to weight screening, or reluctance to discuss the topic of overweight with adolescents and their parents. Twenty-five nurses volunteered to recruit, and became responsible for issuing information and obtaining informed consent from participants and their guardians. We intended to match the participants in the intervention group with controls from the same schools; however difficulties in recruiting sufficient controls, made it necessary to extend the number of schools. At post-test (T1), reminders were given to the participants in advance, both by SMS and directly by the school nurse. A second appointment for T1-test was made whenever participants did not show up. The sample size of the original protocol was estimated to 96 in each group based on an effect size of 0.5, a power of 0.80, a level of significance 0.01 and the use of a two-sided t-test for statistical analysis. A 5% significance level reduced the sample size to 64 per group.

### Outcome measures

#### Primary outcome

Cardiorespiratory fitness was measured with the 20-meter shuttle run test (20mSRT). This reliable and valid test is widely used to assess cardiorespiratory fitness in children and adolescents [35]. The participant is required to run 20 m shuttles back and forth at an audible signal with a starting speed of 8.5 km · h^−^^1^. The pace continues to increase by 0.5 km · h^−^^1^ every minute thereafter with each new pace representing a higher level. The test ends when the participant has to stop because of fatigue, or fails to maintain the pace for two consecutive shuttles. In accordance with the literature, performance was calculated and presented as total shuttle count and end running speed [36], [37], of which the latter was included in the analyses.

#### Secondary outcomes

Health-related quality of life was assessed using the Norwegian version of KIDSCREEN, a generic instrument focusing on physical, mental and social dimensions of well-being measured from the adolescents' perspective [38]. For the present study, a global HRQoL score was constructed based on an index from the 10-item version as described in the KIDSCREEN manual [38]. The timeframe of the questionnaire refers to the last week prior to assessment and consists of the following items: (1) felt fit and well, (2) felt full of energy, (3) felt sad, (4) felt lonely, (5) had enough time for yourself, (6) have been able to do the things that you want in your free time, (7) parent(s) treated you fairly, (8) had fun with your friends, (9) got on well at school and (10) have been able to pay attention. All items were rated on a five-point Likert scale ranging from never to always or not at all to extremely. The scales for negatively worded items were reversed. The raw scores were transformed linearly to a 0–100-point scale, with 100 indicating the best quality of life and 0 the worst [38] The Norwegian version of KIDSCREEN-10 has previously shown satisfactory validity and reliability [39]. Cronbach's alpha of 0.79 at T0 indicated satisfactory internal consistency for the instrument in this study.

Physical activity was self-reported and measured with a single item adapted from World Health Organization Health Behaviour in School-Aged Children (HBSC) surveys [40], validated in the Young-HUNT study [41]. The question was “Apart from the average school day, how often do you play sports or exercise to the point where you breathe heavily and/or sweat?”. The seven response alternatives ranging from “every day” to “never” were recoded into three categories so that “low activity” represented “one day a week or less”, “moderate activity” represented “2–3 days a week” and “high activity” represented “four days a week or more” [41].

*Self-determined motivation towards physical activity and exercise* was assessed using the Behavioral Regulation in Exercise Questionnaire-2 (BREQ-2). The scale consists of 19 items relating to the five types of motivation identified by self-determination theory (from least to most self-determined: amotivated, extrinsic, introjected, identified and intrinsic regulation). A relative autonomy composite score representing self-determined motivation was calculated consistent with past work [42]. The score ranges from −24 to +20 with more autonomous motivation being indicated by higher positive scores. A confirmatory factor analysis has been performed on the baseline results showing adequate factorial validity of the questionnaire [14]. Acceptable alpha coefficients were found for all subscales (0.71–0.86).

*Body image* was assessed using a Norwegian body image scale [43] consisting of four items; (1) I would like to change a good deal about my body, (2) by and large, I am satisfied with my looks, (3) I would like to change a good deal about my looks, and (4) by and large, I am satisfied with my body. The adolescents responded to one of the following categories: does not apply at all; does not apply well; applies somewhat; applies fairly well; applies well and applies exactly. The two negatively formulated items were recoded so that higher scores indicated a more positive body image. All four items were summed to construct a body image score ranging from zero to 20 with higher scores indicating a more positive body image. Previous research has found that the scale has acceptable reliability [44]. In this study, Cronbach's alpha for the scale was 0.87, showing good internal consistency.

*Body mass index* was calculated based on weight and height measurements. Body weight was measured to the nearest 0.1 kg with a portable digital weight. The adolescents wore no shoes and only light clothing. Weight was corrected (−0.5 kg) for clothes. Height was measured to the nearest 0.1 cm with a stadiometer. Age- and gender-specific BMI cut-off values proposed by the International Obesity Task Force were used to categorize the adolescents as overweight or obese [45].

### Procedures

The intervention group received the 12 week-intervention described below, while the participants in the control group were given follow-up as usual by the school health service. Such follow-up consisted of opportunities to meet with the nurse on the adolescent's request and in some cases participation in weekly exercise groups run by the school health service. These groups were offered as an alternative to organized sports. However, the groups did not included more than merely organized exercise comparable to any other typical leisure time sports activity.

Participants in both groups completed self-report measures, measurements of height and weight and a standardized fitness-test at baseline (T0) and after 12 weeks (T1). All measurements took place during school hours. The researchers were responsible for the assessment. The school nurse and the researchers were available to clarify the items in the questionnaires if necessary. The fitness-test was performed in the school's gym, with only one of the researchers present. Standardized information about how to perform the test was given individually. The participants wore running shoes and light clothing.

### The intervention: Young & Active

**Figure 1:**
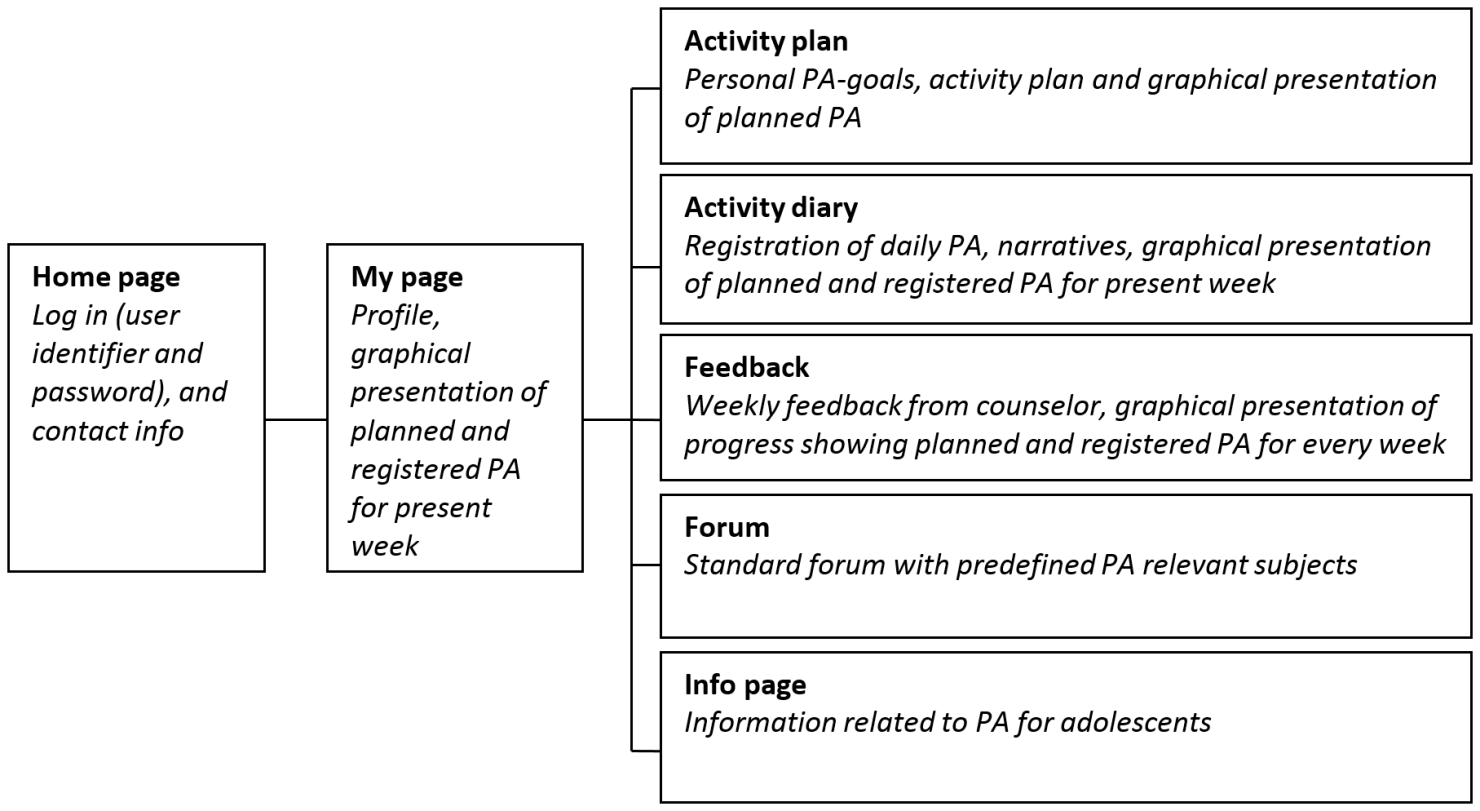
Main content and interactive features of Young & Active [46].

Young & Active was developed with the aim of motivating overweight adolescents to increase and maintain PA and thereby enhance their fitness and HRQoL. The intervention focused on physical activity, how active the adolescents aimed to be, and how they could make self-determined choices to increase activity throughout the day. Weight reduction was not emphasized. The development and the content of the Internet program, as well as an evaluation of its usability, have been thoroughly described elsewhere [46]. In brief, the program offered the participant opportunities to establish personal goals and a plan for physical activity, to register physical activity, to keep a physical activity diary and to get support from a forum. It provided continuous graphical response on progress, frequently updated information on physical activity and, most importantly, weekly individualized feedback and counseling from a health professional. Additionally, the program contained a mailing system with a message box present on every page making it possible for the participant and counselor to exchange short messages independently of the diary and weekly feedback if necessary. A summary of the main content and the interactive features of the program are shown in Figure 1. A simple password-protected page for the counselor was also designed. On this page, the counselor could view a summary of each participant's registration and diary notes, and write and send weekly feedback and monitor the forum.

The intervention started with an individual meeting with one of the researchers. Embedded in the principles of MI, the informal conversation focused on feelings, thoughts and experiences with different forms of sports and physical activities. Additionally, the participant was given access to the program with a brief introduction to it. The following Monday, the participant received a reminder via SMS to start registrations. Except for the first face-to-face introductory meeting, all communication between the participant and the counselor took place online. One of the researchers (a physiotherapist trained in MI) provided the written counseling to every participant every Monday for 12 weeks. The key principles from the application of autonomy support with examples from MI-principles and the counselors' feedback are given in Table 1. The participant was encouraged to make daily registrations and write narratives on PA and exercise. However, the program allowed for backdating within the same week, so that registrations did not have to be made every day. Daily registrations and writing in the diary took an estimated 5–10 minutes. If registrations were missing for the entire week, the participant received a standardized reminder via SMS and a standardized message in the program.

### Ethical aspects

The study was reviewed and approved by the Regional Committees for Medical and Health Research Ethics in Norway. We emphasized that our informants were given sufficient and understandable information about the study, the purpose, the right to withdraw, potential harms and benefits. All participants gave written assent to participation by signing a form, and at least one of their guardians signed an informed consent. Based on a rigorous risk analysis, appropriate steps were taken to ensure program security and thus security and anonymity of the participants. During the development and preliminary implementation of the study, several ethical issues became apparent. For this reason, a thorough ethical evaluation of the entire intervention was carried out and published [32]. It seems evident that the ethical aspects of the present intervention were mainly concerned with the vulnerability of adolescents being identified as overweight. However, we argue that the individually tailored feedback as provided in this intervention had a unique potential to empower the participant to make decisions about his or her own health behaviour. The present study is registered at ClinicalTrial.gov (NCT01700309).

### Statistical analyses

Due to a limited number of cases and a relatively small amount of missing values, no imputation of missing values was considered necessary. No sum scores were computed if one or more items were missing. All participants were analyzed in the group to which they were allocated. Distributions of all continuous variables were visually inspected. Data were generally skewed, thus data are described with median, minimum and maximum values, and the groups compared with non-parametric methods (Chi-square test and Mann-Whitney Wilcoxon test). The small sample size did not allow for multivariate analysis. To compare pre-to post intervention differences within groups, a paired samples t-test was used as the mean differences followed normal distribution. Crude differences between groups were analyzed with an independent samples t-test. The differences between change in the intervention group versus the control group are expressed as effect sizes calculated with Cohen's d and categorized as <0.5 =  small effect, 0.5–0.8 =  medium effect and >0.8 =  large effect [47]. In addition, we investigated if frequency of use affected the outcomes. We chose a cut-off at seven weeks and performed analyses comparing those who made registrations for more than seven of 12 weeks (“frequent intervention users”) and the control group. Finally, logistic regression was performed to assess the impact of factors on the likelihood of being a frequent user versus an infrequent user of the intervention. The results are expressed as odds ratio (OR) with 95% confidence interval (CI). P-values ≤0.05 were considered statistically significant and all tests were two-sided. The analyses were performed using SPSS© 21.0.

## Results

### Participants

**Figure 2:**
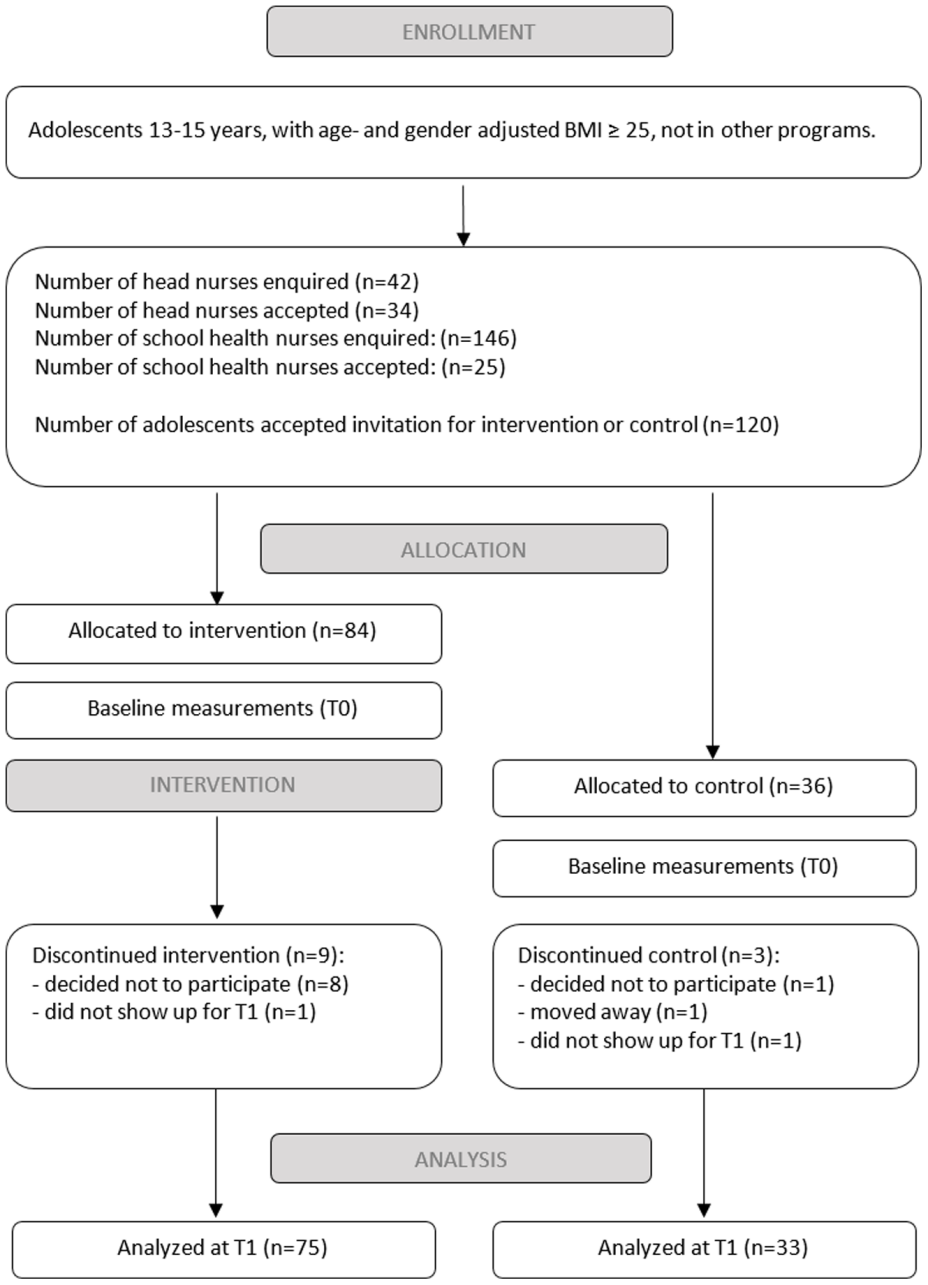
Study design and participant flow.

In total, 84 and 36 adolescents agreed to participate in the intervention and control groups respectively (Figure 2). Only eight of 84 adolescents withdrew from the intervention group during the intervention period leading to an attrition rate of 9.5%. The most common reason for resigning was that participation was felt to be too stressful or time-consuming. In addition to those who actively resigned, one participant in the intervention group never appeared for retest, for reasons unknown. Among the control participants, three discontinued participation. Eight of the 12 who discontinued participation were boys. There were no statistically significant differences between the discontinuers and the continuers on any of the study variables.

Participant characteristics at baseline (T0) and post-intervention (T1) are presented in Table 2. More intervention participants (6) than control group participants (1) became normal weight (age- and gender adjusted BMI <25) during the intervention. In addition, four intervention group participants went from being obese to overweight, while two participants from each of the two groups went from overweight to obese during the intervention period. About one third, 36% of the participants in the control group and 31% in the intervention group reported an increased PA level (from low to moderate, low to high or moderate to high) at T1. Table 3 displays median and minimum and maximum values for assessments of all study variables at both assessment points. At baseline, there were no statistically significant differences between the intervention- and control groups, except for self-determined motivation for PA and exercise as indicated by the relative autonomy index (BREQ2), which was significantly higher in the control group (*P* = 0.04).

**Table 2:** Participants' characteristics.

	T0		T1	
Characteristics	Intervention	Control	Intervention	Control
	n = 84	n = 36	n = 75	n = 33
Age, median (min/max)	13.70 12.9/15.1)	13.78 (12.8/15.0)		
Girls	50 (60%)	23 (64%)	47 (63%)	22 (67%)
Born in Norway	72 (86%)	29 (81%)	63 (84%)	26 (79%)
Overweight	57 (68%)	23 (64%)	46 (61%)	19 (58%)
Obese	27 (32%)	13 (36%)	23 (31%)	13 (39%)
Normal weight			6 (8%)	1 (3%)
Low activity	25 (30%)	8 (22%)	13 (17%)	6 (18%)
Moderate activity	41 (49%)	21 (58%)	37 (50%)	13 (40%)
High activity	18 (21%)	7 (19%)	25 (33%)	14 (42%)

**Table 3:** Median and minimum/maximum values for primary and secondary outcome measures at baseline (T0) and post intervention (T1).

	T0		T1	
Assessments	Intervention	Control	Intervention	Control
	n = 84	n = 36	n = 75	n = 33
**20mSRT** (km/h)	9.50	9.50	10.00	9.50
	(8.5/12.0)	(8.5/11.0)	(8.5/12.0)	(8.5/11.5)
missing	n = 0	n = 0	n = 8	n = 1
**Shuttles**	23.0	20.0	25.0	17.5
	(7.0/64.0)	(6.0/51.0)	(7.0/72.0)	(6.0/55.0)
missing	n = 0	n = 0	n = 8	n = 1
**KIDSCREEN-10** (0–100)	67.50	68.75	68.75	70.00
	(17.50/100.00)	(30.00/92.50)	(25.00/97.50)	(40.00/92.50)
missing	n = 9	n = 6	n = 7	n = 1
**Body image** (0–20)	7	8	10	8
	(0/17)	(1/19)	(0/20)	(0/19)
missing	n = 3	n = 3	n = 4	n = 2
**Relative autonomy index** (−24–20)	7.92	10.83	8.75	10.25
	(−11.00/19.33)	(−2.50/17.83)	(−9.00/18.67)	(−3.33/17.83)
missing	n = 2	n = 2	n = 6	n = 0
**Body mass index** (kg/m^2^)	26.62	27.45	26.40	27.45
	(22.09/37.79)	(22.37/36.36)	(21.59/40.04)	(22.33/36.37)
missing	n = 0	n = 0	n = 0	n = 0

### Within-group analysis

A paired samples t-test was conducted to evaluate changes on the outcome variables within groups. The results are presented in Table 4. Intention-to-treat analysis showed a small, however statistically significant increase in cardiorespiratory fitness for the intervention group (*P* = 0.01). In addition, as opposed to the control group, HRQoL as measured by KIDSCREEN-10 increased significantly in the intervention group (*P*<0.01), as did body image (*P*<0.01). Conversely, there was a statistically significant increase in BMI in the control group during the test period (*P* = 0.02), while no change was seen on BMI in the intervention group. Self-determined motivation measured by relative autonomy index did not change in any of the groups.

**Table 4:** Mean differences for the primary outcome measure and secondary outcome measures within groups, confidence intervals (CI) and *P*-values.

Outcome measure	N	Mean diff.	95% CI	*P*-value^a^
		T0 to T1		
**20mSRT^b^**				
Intervention	67	0.14	0.03;0.25	0.01
Control	32	0.00	−0.08;0.08	1.00
**KIDSCREEN-10**				
Intervention	61	4.59	2.08;7.10	<0.01
Control	28	−0.63	−4.05;2.80	0.71
**Body image**				
Intervention	68	1.57	0.66;2.49	<0.01
Control	28	0.29	−0.68;1.25	0.55
**Relative autonomy index**				
Intervention	67	0.03	−1.24;1.30	0.97
Control	31	−0.21	−1.85;1.44	0.80
**Body mass index**				
Intervention	75	−0.10	−0.31;0.10	0.32
Control	33	0.29	0.06;0.53	0.02

a *P* values for paired samples t-tests

### Between-group analysis

Differences in between-group changes are presented as effect sizes in Table 5. A small effect (0.14; 95% CI [0.01;0.28]; *P* = 0.04) was found between the groups on CRF after the intervention and a moderate effect (5.22; 95% CI [0.90;9.53]; *P* = 0.02) was found on HRQoL. Moreover, a moderate effect was found on BMI (−0.39; 95% CI [−0.74;−0.05]; *P* = 0.03). The remaining differences were not statistically significant and the effect sizes small and non-existent. Analysis including controls (n = 33) and frequent users (n = 35) of the intervention revealed moderate effect sizes on CRF (*P* = 0.04), body image (*P* = 0.04) and BMI (*P* = 0.01). However, the effect size on HRQoL was smaller (4.21; 95% CI [−0.74;9.15]; *P* = 0.09) when comparing frequent users to controls, than in the analysis including the complete intervention group.

**Table 5:** Between-group differences and effect sizes after the intervention (T1).

	Intervention versus control				Frequent users versus controls			
Outcome measure	Meandiff	95% CI	*P*-value^a^	ES^b^	Mean diff	95% CI	*P*-value^a^	ES^b^
*Primary*								
20mSRT	0.14	0.01;0.28	0.04	0.39	0.21	0.01;0.41	0.04	0.55
*Secondary*								
KIDSCREEN-10	5.22	0.90;9.53	0.02	0.56	4.21	−0.74;9.15	0.09	0.45
Body image	1.29	−0.26;2.83	0.10	0.40	1.68	0.10;3.26	0.04	0.56
Relative aut.index	0.23	−1.92;2.39	0.83	0.05	0.51	−1.86;2.88	0.67	0.11
BMI	−0.39	−0.74; −0.05	0.03	−0.50	−0.52	−0.89; −0.16	0.01	−0.70

a *P*-values for independent samples t-tests

b Cohen's d

### Frequent- or infrequent use of the program

Logistic regression was performed to assess the impact of selected variables on the likelihood of being a frequent (n = 35) or an infrequent user (n = 41) of the intervention. Each study variable was tested individually against frequent/infrequent use. Variables that were statistically significant in univariate analyses were entered into a multiple regression model. In this model containing HRQoL, BMI and self-determined motivation, HRQoL and BMI remained independent predictors of frequent use. For each extra unit on KIDSCREEN-10, the participants were 9% more likely to use the intervention frequently (*P*<0.01; OR = 1.09; 95% CI [1.03;1.16]), while for each unit increase in BMI, they were about 20% less likely to use the program frequently (*P* = 0.03; OR = 0.81; 95% CI [0.68;0.97]). CRF was not included in the model as we expected this variable to be heavily confounded by BMI. Gender was not associated with frequency of use.

## Discussion

To our knowledge, this is the first study to investigate the effects of an individually tailored Internet intervention using principles from SDT and MI to increase CRF and HRQoL among young people with overweight and obesity. The results indicate that we were successful in reaching the group approached. Our findings support that during the 12-week intervention, we managed to influence the intervention group participants' CRF, HRQoL and BMI in a positive direction. The short-term effect on the primary outcome CRF was significant, but modest; however some factors should be considered which might lend more weight to the result. First, the present intervention emphasized self-determined physical activities and exercise in general, not just endurance activities. Enhancement of CRF demands systematically increased efforts in aerobic activities over time. Thus, increased CRF in this study depended on the participant understanding the information provided by the intervention, engaging in aerobic activities and possessing or acquiring self-regulatory skills to execute planned PA with sufficient intensity [31]. We have to take into account that for different reasons, some intervention group participants perhaps chose less intensive activities and thus did not contribute to increasing the total CRF. Secondly, our sample had notably low initial CRF. Compared to centile curves from a general sample in an English study [37], both girls and boys in the present study scored below the 25^th^ percentile [14]. Despite substantial evidence demonstrating that CRF is a powerful marker of health [9], there are no agreed definition or cut-offs for low CRF in youth. However, studies on adults indicate that individuals gain the largest benefits to health by moving out of the lowest quartile or quintiles for CRF [48]. Hence, we argue that any improvement in a positive direction is of value to the group of overweight and obese adolescents and might be the beginning of a positive development towards higher levels of CRF. Excess weight was most certainly a strong contributor to the low CRF of the present sample. The inverse relationship between BMI and CRF is well documented [8]. However, since there were only minor changes in mean BMI in the intervention group and minor changes in CRF in the control group, it seems unlikely that BMI can explain the entire between-group difference in CRF post intervention.

We were unable to detect changes in the amounts of leisure time PA in favor of the intervention group by means of the single item measuring PA frequency. Hence, there are reasons to assume that increased CRF within this group was caused by an increase in PA intensity and possibly duration. This relates well to the intervention's content, features and counseling, which explicitly emphasized the importance of increasing intensity in endurance activities to enhance aerobic capacity (Table 1). It is important to notice that since we included only assessment of cardiorespiratory fitness, we were not able to document potential improvement on, e.g. musculoskeletal or motor fitness.

We also observed effects of the intervention on well-being, conceptualized as HRQoL. A large body of research has found that overweight and obese youth report lower health-related quality of life than do adolescents with normal weight, and the physical dimension seems to be one of the most affected [3], [49]. Compared to a representative Norwegian sample of adolescents, the participants in the present study reported a lower baseline HRQoL [39]. KIDSCREEN-10 does not allow for analyses of dimensions of HRQoL. Even so, the fact that a generic short form instrument captured improvement in overall HRQoL following an intervention focusing on PA, may emphasize the importance of physical performance and participation on well-being for this group.

Additionally, the intervention had a beneficial effect on BMI, mainly reflecting a BMI gain prevention among the intervention participants. While the intervention group had a small non-significant reduction in BMI, the control group increased significantly. Findings from studies indicate that adiposity continues to increase in obese children who do not receive treatment [50], [51]. Thus, a stagnation following an intervention must be considered a goal in secondary preventive interventions. When comparing frequent users of Young & Active to the controls, we found an even larger effect size, pointing to a relationship between adherence and the outcome and an additional effect on BMI reduction for intervention users. The intervention did not emphasize weight reduction or eating behavior. However, previous studies on adults have documented that interventions targeting one specific health behavior, can have motivational impact on other health behaviors [52]. Even though the intervention focused on PA and exercise, the participants may have been influenced to reduce their sedentary behavior, and change diet and eating patterns as well.

We also hypothesized that the intervention would improve body image. When all participants were included in the analysis, the effect size on body image was insignificant and small. However, we found a moderate per protocol between-group effect when including only those who used the intervention frequently. This may indicate that the intervention, depending on the use, managed to facilitate body satisfaction, however, the difference is too small to be of particular clinical importance.

Even though the intervention aimed to influence self-determined motivation specifically, within-group analyses revealed no statistically significant increase in the intervention group on regulation of motivation, and no between-group effect was found post intervention. Both groups voluntarily signed up for an intervention study with an explicit focus on PA. Thus, it is likely that they were all more self-determined in their self-regulation of motivation for PA and exercise than the general population of overweight and obese adolescents, an assumption supported by their relatively high positive baseline relative autonomy index-scores. Another explanation is that even though we emphasized autonomy by including need-supportive features in the intervention, the young participants may have perceived their motives for taking part as somewhat controlling. As a result, some may have felt the pressure to become more physically active, which contributed to increasing controlled forms of regulation and evened out a potential increase in autonomous regulation. This study does not present data on the different forms of regulation, however previous studies have found introjected regulation to relate positively to autonomous forms of motivation [53], [54].

Finally, this study adds some information about the probability of using the intervention by showing that higher initial BMI reduced, while higher initial HRQoL increased, the likelihood. Even though most of the withdrawers were boys, gender did not seem to be a significant factor for intervention use. These findings will be supplemented with an upcoming detailed process evaluation.

### Strengths and limitations

#### Strengths and limitations of the intervention

We systematically developed the Young & Active based on theory and in cooperation with representatives from the target group, which is likely to have influenced use and satisfaction [46]. Among the many advantages of Internet interventions is accessibility, anonymity and flexibility for the users. In addition, the health promoters are given improved opportunities for maintenance and updating of the intervention. The present intervention included multiple active ingredients with automated feedback and individually tailored need-supportive counseling, all of which are factors shown to improve exposure [18], [55]. However, we have to take into consideration that for some, the intervention might have been too extensive.

A great disadvantage for an Internet intervention such as ours is its limited lifetime. From the time Young & Active was planned, technology and new innovative applications have developed at a furious pace. Although the program's design and functions already may seem outdated, the content and characteristics will make a valuable basis for further development and improvement.

#### Strengths and limitations of the study

Application of pretests and a control group design is among the appreciable strengths of this study. The small pretest differences between the groups decreased the likelihood of initial selection biases. As shown, with only one exception, there were no significant differences between the groups on any of the baseline variables and thus no need to adjust for possible confounders in multiple analyses. One of the most substantial problems in web-based prevention is high attrition rate and low use of the interventions. In the current study, the actual attrition rate was low, and even though half of the intervention group made only infrequent registrations, these participants may have been exposed to intervention content despite not using the diary. Lastly, we chose a clinically realistic way of recruiting the sample. This provided valuable information about pitfalls and possibilities for future potential large-scale assessments and implementation.

Randomized controlled trials are generally more reliable than quasi-experimental trials. Hence, a major disadvantage of the current study is its lack of randomization. The vulnerability of the target group and the clinical setting, made it unethical to assign participants randomly. The individuals were included sequentially and based on convenience, with the consequence of potential between-group baseline differences in unmeasured variables. Recruitment of participants can be a significant obstacle in research, as it proved to be in the present study. In this case, the greatest hinder turned out to be that the majority of the school nurses, on whom we relied to engage participants, declined our requests for assistance. The consequence was an extensive recruitment process, which due to time limitations had to be terminated before we had two equally large groups containing the number of participants suggested by the power analysis. The small sample size did not provide sufficient power to analyze subgroups like gender. In addition, the generalizability of the study was heavily reduced, both because of the quasi-experimental design, and because of the lack of information about participants who were invited, but declined participation. Although the two groups were included and assessed during the same months, they participated during two following school years. Potential seasonal variations between the two years may have affected the PA level between the groups. As in all research, there are validity issues associated with the use of self-report measures. An objective measure of PA would have increased the validity and added valuable information to the analyses. Another disputed assessment undertaken in this study, is BMI, which does not take full account of maturation status. In addition, the reliability and appropriateness of the 20mSRT have been discussed in the past, with motivation, competency and perceived worth being mentioned as variables that influence performance. However, in a recent systematic review, the authors concluded that the test was valid and reliable for estimating CRF in children and adolescents [56]. We used a sum score from the 10-item version of the KIDSCREEN questionnaire. The results might have been different if we had used the extended versions (27- and 53-items) which include different sub-dimensions. Finally, this study does not generate evidence for long-term effects, which is an important limitation. One-year-follow-up data are being obtained, and will be analyzed and presented in the near future.

## Conclusion

Our results give preliminary support to the efficacy of an Internet intervention for increasing CRF and HRQoL in adolescents with overweight and obesity. We also found beneficial effect on BMI. Despite national guidelines for measurement and follow-up of overweight and obesity, our impression is that face-to-face health-behavior counseling of overweight and obese adolescents in primary health care is limited or non-existent. The integration of Internet technology into primary care practice offers new possibilities for interaction with the target group with several advantages for the users as well as for the health promoters. The findings of the present study give promise to future interventions applying newer technology in a primary health care setting.

## Supporting Information

Protocol S1 **Trial Protocol.**
(PDF)

Checklist S1 **TREND Checklist.**
(PDF)
