# Supplementary material for: The Outcomes of a 12-Week Internet Intervention Aimed at Improving Fitness and Health-Related Quality of Life in Overweight Adolescents: The Young & Active Controlled Trial
Source: PLoS One. 2014 Dec 5;9(12):e114732. doi: 10.1371/journal.pone.0114732 (PMC4257715; doi:10.1371/journal.pone.0114732)
Supplement: Protocol S1 — Trial Protocol. (PDF) [file pone.0114732.s001.pdf]

“Young fit and happy”

“Meaningful physical activity as means to improve quality of life and prevent weight gain in over-weight and obese adolescents.”

The overall purpose of this study is to establish an effective intervention aimed at enhancing meaningful physical activity and quality of life in overweight and obese adolescents by establishing an activity plan and give individualised web-based counselling. Further, the purpose is to better understand how overweight and obese adolescents experience being exposed to a web-based intervention program. To participate in an intervention puts demands on the adolescents to change their lifestyle in order to reach their goals. The need for more comprehensive knowledge about what works according to weight and fitness, what motivates the adolescents to change and finally how adolescents experience interventions that require lifestyle changes is needed.

Until now, no single intervention has proved to be effective in treating and/or preventing overweight and obesity in adolescents. However, a systematic review on treatment of obesity shows that combined behavioral lifestyle interventions compared to standard care or self-help can produce a significant and clinically meaningful reduction in overweight children and adolescents (Oude Luttikhuis 2009). Another systematic review focusing on prevention strategies showed that combining dietary and physical activity approaches did not significantly improve BMI, but some studies that focused on dietary or physical activity approaches showed a small but positive impact on BMI status. Nearly all studies included in the review resulted in some improvement in diet or physical activity. (Summerbell et al 2009). Even though many of the programs have little impact on BMI, the increase in physical activity and improved diet is shown to have a positive impact on the adolescents’ health, which is a desirable outcome in itself (Blair et al 1999). In “Young, fit and happy” it is our assumption that increased physical activity improves the adolescents’ fitness (health) and their quality of life. The challenge is to get the adolescents’ motivated for lifestyle changes in order to make them permanent. Focusing on how the adolescents are doing through the process and how they experience being in the process of an intervention should give us valuable knowledge on how to design effective theory-based programs for treating and preventing overweight and obesity. Therefore the purpose of this study is twofold, to test a web-based intervention by a randomized controlled trial and to gain in depth knowledge on the adolescents’ experiences of being overweight and being exposed to a web-based intervention program. In a review, web-based weight management programs for overweight children and adolescents show a big potential for weight management. However, more research is needed to investigate types of web-interventions appropriate for boys and girls in this age group, and further evaluate the process and short- and long-term effects of such interventions” (An Y et al, 2009).

Background

The prevalence of overweight and obesity is increasing in both adult and child populations throughout the world (WHO 2006). Obesity is seen as one of the most important public health threats because of the significant impact of chronic conditions associated with obesity. Obesity in adults increases the risk of cardiovascular diseases, diabetes, and musculoskeletal disorders (Must et al 1999). Obesity is not as strongly associated with morbidity in adolescencehttp://archpedi.ama-assn.org/cgi/content/full/158/1/27 - REF-POA20221-4#REF-POA20221-4, but is a strong precursor of obesity and related morbidity in adulthood, with 50% to 80% of obese teenagers becoming obese as adults (Berensson et al 1998, Singh et al 2008). During adolescence, overweight and obesity are often a burden that results in psychosocial problems, poor quality of life and a reduced capacity for physical activity (Hill & Lissau 2002, Molnar & Livingstone 2000, Ravens-Sieberer et al 2001, Fallon et al 2005, Stern et al 2006, de Beer et al 2007). Adolescence is a critical period for the onset of obesity and for obesity-associated morbidity in later life. Therefore, it is important to increase our knowledge and develop effective interventions to prevent and treat over-weight and obesity in adolescence.

Preventing and treating overweight and obesity. Overweight and obesity arise from an imbalance between energy intake and expenditure. By adolescence the problem is particularly intractable if overeating is coupled with a lack of physical activity (Whitaker et al 1997). Cochrane reviews have looked upon the effects of treatment and prevention of overweight and obesity in childhood and adolescence (Summerbell et al 2003+2005+2009, Oud Luttikuis 2009). They conclude that so far we lack good quality studies and it is not possible to draw sound conclusions on the most effective interventions related to childhood obesity. However, a comprehensive strategy that addresses dietary and physical activity change, together with psychosocial support and a family focus is recommended (Summerbell et al 2005, Artebaum 2006), and the need for further research is obvious. Adolescents are more concerned about the psychosocial than the physical consequences of their overweight (Booth et al 2008). The psychosocial consequences being ie bullying, social exclusion, inability to participate in mutual activities with peers, leading to low self-esteem and low quality of life (Booth et al 2008, French et al 1996, de Beer et al 2007).

Interventions aimed at enhancing physical activity.
Bodyweight is regulated through the interaction of genetic propensity and habitual lifestyle. Genes gives individual disposals for obesity, environment and habitual lifestyle determine if they actually become obese (McArdle et al 2001). According to research findings, the energy intake in Norway has remained stable over the last decades (SEF 2000). It is likely that an increase in body weight can be explained by decreasing daily physical activity due to structural changes in society (Hill & Peters 1998). It is shown that physical activity diminishes from early childhood into adolescence (Sallis et al 1992, Stone et al 1998). Several investigations also point to a lower level of habitual physical activity among persons with overweight problems compared to normal-weighted persons (McArdle et al 2000; Stone et al 1998). Efforts to decrease sedentary activity in youth will affect the incidence of obesity and future physical activity and will reduce future health risks (Douthitt 1994, Kien & Chiodo 2003, Rising et al 1994). Blair et al (1999) have shown that the advantages of being fit are striking and that people can be fit even if they are overweight or obese, and thus they have lowered risk of disease. Therefore, interventions should emphasize increased habitual physical activity more than individual body weight. Unfortunately, the currant emphasis on competitive community sports can act as negative enforcement for increased physical activity by some adolescents, especially those who are clumsy or overweight (Epstein et al 1991,Sallis et al 1992). Therefore, consideration should be given not only to the environments of communities that can promote satisfying physical activity but also to our overall societal outlook toward activity for all individuals, not just for the persons with exceptional athletic competence. In the general population of adolescents, sedentary activity could be replaced by a great variety of physical activities that could increase daily energy expenditure. An important step in behaviour change may be to substitute some physical activity for periods of the day when there is none. This can be activities chosen for pleasure or purpose, but in our view, an individual intervention program should focuses on providing fun physical activity that even unskilled, inactive adolescences can enjoy. It is shown that overweight adolescents endorse extrinsic weight related goals for physical activity, whereas intrinsic goals positively predicts self-determined motivation, quality of life and exercise behaviour (Gillison et al 2006). Motivation is a key factor in the development of successful interventions. Thus, it is important to develop an intervention to enhance physical activity that is perceived as meaningful for the adolescents.

New means of communication, such as blogs and chatting on the internet have become widely available and provide excellent opportunities for low threshold counseling using feedback to guide and support over-weight and obese adolescents. Adolescents are familiar with and use the internet frequently to communicate with others. Recent research suggests that internet technology is a promising way to change adolescents' health behavior (An Y et al 2009).

Quality of life. Over the last decade, quality of life has emerged as an important concept in evaluating health care with respect to prevention, treatment, and rehabilitation. The concept is based on several key ideas. One idea is that quality of life is a subjective phenomenon in that all individuals have their own perspective concerning their well-being and their lives. Moreover, when used in a medical

context (health-related quality of life, HR-QOL), quality of life is generally conceptualized as a multidimensional construct encompassing domains such as physiological, mental, social, and spiritual areas of life (Eiser et al 2001, Pal 1996, Spilker 1990). HR-QOL measures often consist of multiple items grouped together into various domains of health and functioning. This multidimensionality provides researchers and clinicians with information about the impact of disease, or the effect of various interventions on different aspects of quality of life, and serves as a framework for identifying and developing strategies to promote quality of life (Conolly and Johnson 1999). As overweight and obesity affects both physical and psychosocial areas of life an intervention aimed at promoting health related quality of life seems appropriate. In a qualitative study adolescents say that quality of life is about the positive cycles of life (Helseth and Misvær 2010). Feeling good, being satisfied with oneself and having an overall positive attitude are in most cases described as the starting points of a positive cycle. In order to get into and stay in the positive cycle, a positive self-image, good friends and good family relations are important. Consequently, adolescents' quality of life is threatened when these factors are negative. Friends seem to be the most significant factor. Self-image and popularity are factors associated with the ability to get friends (Helseth and Misvær 2010).

Aim and hypothesis

The overall purpose of the study is to examine the extent to which a web-based intervention influences physical activity, fitness and quality of life in over-weight and obese adolescents Further, the aim of this study is to explore and describe how adolescents experience being over-weight and obese, what they perceive as meaningful physical activity and finally how adolescents experience lifestyle changes as demanded in the intervention study.

Prevalence and sample characteristics

For adults, BMI (Body Mass Index; weight in kilograms divided by the square of height in meters) values at or above 25 indicate overweight and a BMI at or above 30 defines obesity. No such generally accepted definitions exist for children and adolescents. However, international standardized cut points have been proposed, among others by Cole et al (2000) and experts support the use of BMI cutoff points for children. The cut points proposed by Cole et al (2000) are widely used and take age, gender and sex into consideration in the estimation. An example of cutoffs for overweight (BMI 25 in adults) and obesity (BMI 30 in adults) in 13 year old adolescents are shown in the table below (Cole, from Nowicka & Flodmark 2006):

Age	BMI (25)<br>Boys<br>Over-weight	BMI (25)<br>Girls<br>Over-weight	BMI (30)<br>Boys<br>Obese	BMI (30)<br>Girls<br>Obese
13	21.91	22,58	26,84	27,76

The prevalence of overweight and obesity of 8 and 12 year old children in Oslo, using Cole's index and based on objective measures of weight and height, was 21 % (Vilimas et al 2005). In another study in Oslo, based on self-reported measures of weight and height, the prevalence was 12% among 15 year old boys and 7 % among 15 year old girls (Lien et al 2007), which is in accordance with the prevalence of self-reported data in several other European countries (Lissau et al 2004). It is shown that the prevalence of overweight might be underestimated by as much as 50% when based on self-reported data (Raben et al 2000). Thus, it is important to get more objective data on weight and height in order to see the true prevalence of the condition and design studies on overweight and obesity using objective measures.

Target group

The present study focuses on adolescents in 7th grade living in Oslo. Since overweight is over-represented in parts of Oslo where the majority of the inhabitants are non-western, it is important to include adolescents with different cultural background and a variation in socioeconomic conditions (Vilimas et al 2005, Lien et al 2007). The target group is overweight adolescents, meaning their BMI is over or equal to the 95th percentile (BMI>25) and they should not have severe health problems as a

result of their overweight. The focus is primarily to prevent a negative development, and the aim is that the school health service in the future might implement a modified version of this strategy for working with overweight adolescents. In another study in Oslo, “Big and strong”, the target group is obese children and the focus is on treating obesity and the program is situated in a hospital (Ullevål University Hospital 2008). Yet another study in Oslo is the “HEIA”-project (http://www.heia-prosjektet.org/index.html) which is an intervention study aimed at preventing overweight and obesity among Norwegian children. Young, fit and happy is a study where the target group is in the school health care, the adolescents have a weight problem, but are not being treated medically, and so far very few interventions exist where the intention is to intervene through the ordinary school health service.

Design, sample and an overview of the study

The design of the study is a complex intervention study with a multi-method design, consisting of a randomized controlled trial and a qualitative study. The sample will be drawn from schools in Oslo. Oslo will be divided into four sectors to secure variation on sosio-demographic variables. Overweight adolescents will be randomly selected within each sector and placed in the intervention group or control group respectively. The sampling will be based on objective measures of weight and height and using Cole’s index to define overweight and obesity. Weight and height will be measured by the school nurse and will be coordinated with her regular meetings with the adolescents in 7th grade. New guidelines for weight control will be implemented in Norwegian schools, most likely during 2010. This means all pupils in the 7th grade will have their weight and height measured by the school nurse. The study group will receive the web-based intervention and the control group will receive standard care. At baseline, 6 months and 1 year both the experimental group and the control group will be measured at relevant variables. In the qualitative part of the study, in depth interviews with the adolescents in the intervention group will be performed and they will write daily unstructured blogs on the web. The sampling procedure for the qualitative part of the study will follow the sampling procedure of the intervention study. From the intervention group, a strategic or purposive sample of adolescents will be asked to participate in the qualitative part (Gerrish and Lacey 2006). A sample of maximum variation according to gender and sosio-demographic variables is intended. Sampling continues until saturation is reached and the adolescents will be interviewed in depth before the intervention starts, further at 6 and 12 months after the intervention is ended.

The study.

The intervention will consist of three meetings between the adolescent, the parents and the researchers, and weekly web-based blog-counselling. The intervention period is six months and during the intervention and the follow up period the adolescents will be interviewed three times.

<u>Three Meetings.</u> At the 1st meeting the adolescents will fill in the baseline data and the adolescents that consent to take part in the qualitative study will participate in the first interview. The first meeting will also be an introductory meeting where the adolescent and his/her parents will be informed thoroughly about the purpose of the study as a whole and especially about the intervention. It will be made clear that the goal of the intervention is to increase physical activity, fitness and quality of life through meaningful activities. Weight reduction is not focused; however, it is desired to prevent weight gain through increased physical activity. The 2nd meeting will take place one week later and at this meeting the individual intervention is constructed together with the adolescent and the parents. An activity plan is made up according to the following criteria: The goal of the activities is to increase fitness and the activities should be perceived meaningful by the adolescents. Further the activity plan should be made up according to frequency of the activity (preferred daily); intensity of the activity (increased pulse, increased breath frequency); duration of the activity (1 hour a day); variation between purposeful activity (e.g. walking to school) and joyful activities (e.g. joining a dance class), and finally including some social activity. The second meeting should also prepare for the blog-counselling. The 3rd meeting will take place at the end of the intervention (after 6 months) and the purpose is to evaluate the intervention and motivate the adolescent to continue with physical activity. In addition the adolescent will fill in the questionnaires at this time and those participating in the qualitative study will be interviewed. The same instruments will be filled in at a follow-up after 1 year and the third qualitative

interview with the adolescents will take place. In addition qualitative interviews will be performed with the parents of the adolescents taking part in the qualitative study at the one year follow-up.

Diaries and blog-counseling. A web-based, partly structured diary (blog), on activity and narratives about how their day has been is filled in by the adolescents daily. Personal blog-counselling is given once a week during the first three months, thereafter once a month during the next three months. The counselling will be given within in a frame of self-determination theory, the theory of self-efficacy and quality of life. The purpose of this counselling is to give individualised response to each adolescent and support and motivate them according to their needs.

**Overview of the intervention:**	**Overview of the qualitative part:**
Meeting the adolescent and the parents 1:<br>• Base-line (T0)<br>• Introduction	*Meeting the adolescents1:*<br>• Qualitative in-depth interviews with adolescents (T0)
Meeting the adolescent and the parents 2:<br>• Constructing an activity plan<br>• Introducing the blogs and the chat-room<br>• "Starting up"	
Activity plan is carried out<br>• Blog-counselling and motivation for six months	*During the intervention period:*<br>• Unstructured blog narratives
Meeting the adolescent and the parents 3:<br>• Evaluating<br>• Filling in questionnaires (T1)<br>• In depth interview 2	*Meeting the adolescents 3:*<br>• Qualitative in-depth interviews with adolescents (T1)
Follow up after one year:<br>• Filling in questionnaires (T2)<br>• In depth interview 3	*Follow up after one year:*<br>• Qualitative in-depth interviews with adolescents and parents (T2)

Data collection and measures

The qualitative interviews will follow partly structured interview guides developed according to theory on quality of life, meaningful activity, motivation and earlier research on experiences of being overweight and obese. The interviews will be recorded, transcribed and analysed as text.

In the RCT, at baseline (T0), key aspects of overweight in adolescence will be assessed in relation to physical activity and quality of life (HRQOL). The effectiveness of the individual intervention will be addressed by assessing the key aspects before the intervention (baseline), directly after the intervention (6 months) (T1) and at one follow-up measure (T2). Thus, all the included adolescents complete questionnaires and tests on physical activity, fitness, diet, quality of life, self-efficacy, BMI, exercise motivation and socio-demographic variables. Physical activity will be measured with the relevant part of the Young-HUNT questionnaire (HUNT, 2006), validated for use in adolescences (ages 13 to 18 years) (Rangul et al., 2006). Fitness will be tested with a maximal multistage 20 m shuttle run test (Leger et al., 1988). The adolescents' diet will be considered using a pre-coded food diary, validated for use in a Norwegian adolescent population (Andersen et al 2005). Health related quality of life will be measured with the Norwegian version of Kidscreen (Haraldstad et al 2010), and self efficacy with the widely used 8-item version of Schwarzer's self-efficay scale (Schwarzer 1993). Exercise motivation will be measured by the Exercise Regulations Questionaire (BREQ-2) which will be

translated to Norwegian and validated as part of the study (Markland & Tobin 2004). In addition to the measures at T0, T1 and T2, the adolescents will perform structured activity registrations, write narratives on the blog, and a smaller sample will be interviewed.

Sample

Based on power calculation the sample size in the RCT will be 100 (intervention group) + 100 (control group). The sample size (N) is dependent upon the planned statistical analysis. In this study several complicated analysis will be performed and it is not easy to estimate N without doing a proper power-analysis where all the necessary factors are taken into consideration. This will be done before setting the final sample-size. At this point we give a rough estimate of the needed sample size (Hinkle et al 2003). Given an effect size of 0.5, a significance level at 0.01, power at 0.80 and a two-tailed significance test we estimate N to be 96 in each group (http://hedwig.mgh.harvard.edu/sample_size/quan_measur/para_quant.html). At this point we consider a sample size at 100 (intervention group) + 100 (control group) to be sufficient.

The adolescents eligible for selection to the qualitative study will be approximately 100 (the intervention group). In a qualitative study the sampling plan is evaluated in terms of adequacy and appropriateness (Polit and Beck 2004) Adequacy refers to the sufficiency and quality of data the sample yields and appropriateness concerns the methods used to select the sample. A strategic sampling, within the intervention group, aimed at maximum variation according to gender, ethnicity and sosio-demographic variables seem to be an appropriate sampling procedure here. Thus the sample will consist of overweight and obese adolescents of both genders taking part in an intervention. Sampling continues until saturation is achieved (Polit and Beck 2004), which means when an informational adequacy has been reached. A roughly estimated sample size would be 20-30 adolescents in the qualitative study.

Analysis

The differences between the intervention and control group will be performed using repeated measure analyses for each of the dependent variables in a mixed effect model in SPSS 16. This approach is flexible and it is possible to model the dependence between observations from the same individual. There may also be class and school effects and these can similarly be accounted for. (Fitzmaurice et al 2008). Growth-curve analyses will also be considered given the three different points of measurement.

The structured blogs will be analyzed using descriptive statistics and testing differences in frequency, intensity and duration of the physical activity between the intervention group and the control group (Polit and Beck 2004).

The blog narratives and the qualitative interviews will be analyzed according to Kvale and Brinkmann (2009), implying a hermeneutic approach to data collection and analysis. Hermeneutics is the study of the interpretation of texts and the purpose is to obtain valid understanding and meaning of the texts. Following the guidelines of Kvale and Brinkmann (2009), the analysis will be done within three different contexts: self-understanding, critical common sense, and theory. In the first context, the transcribed interviews will be read and summarized. All interviews will then be re-read and coded, after which categories are assigned using descriptive terminology. The next step will be to interpret the findings by asking questions about meaning within the frame of critical common sense. During the interpretation process, patterns emerge that form the basis for the presentation of the results. In the third context, the findings will be interpreted and discussed in light of the study framework and earlier research.

Research ethics

The adolescents and their parents will be giving informed consent to the study. They are guaranteed full anonymity and confidentiality. The security around the server will assure the safety of the data

given. In the chat room the adolescents will be instructed to use nicknames that only will be known to the researchers.

The primary aim of the networks design is to secure sensitive data. Some very basic measures are taken to ensure that data and identity of the test subjects are protected from any hacks, cracks or attacks – and all sensitive communication is encrypted, and protected with usernames and passwords.

Time Schedule:

2008-2010: Pre study - master students, 2 master thesis in public health completed by June 2010 (UMB).

2010: *Complex intervention study starting*

- Development of the secure web system, with blogs and information pages (in progress).
- Develop the intervention - PhD-student 1, together with the project group (in progress).
- Pilot and start the intervention – PhD student 1 together with research assistant

2011/12: *Measures and interviews*

- Qualitative interviews – PhD student 2
- Ongoing Intervention – PhD student 1 (RCT) and 2 (blogging) and research assistant
- Data analysis starts

2013:

- Finishing the empirical study, continue data-analysis and writing articles and thesis.
- Workshop
- PhD-student 1 completes thesis

2014

- PhD-student 2 completes thesis

This time-schedule is set up according to two doctoral students (PhD-student 1 and PhD student 2) working full time during three years with the study. However, we prefer to have the students work 75% with the study during four years. A research assistant is needed in the intervention period in order to assist with blog counseling. PhD-student 1 (Kirsti Riiser) is working with the RCT and started her work in November 2009. PhD-student 1 is financed through Health and Rehabilitation, The Norwegian Women's Public Health Association (N.K.S.). PhD student 2 will be responsible for the qualitative study. The project leader (Helseth) will have the overall responsibility, will supervise the two PhD-students, co-author all articles and will be responsible for the follow-up at one year. One of the project partners, Hilde Eide will focus more specifically on the communication process during the intervention; ie the relationship between diary blogs, the counselor responses and the health outcomes. In addition, support and cooperation from technological expertise in the development and running of the web-intervention is needed.

Funding applied for:

In this application we apply for funding for one PhD-student (2475000,-); 30% research position for the project leader (744000,-); 50 % position for research assistant (127000,-); 300000,- to develop and run the web-solution for the intervention study; 50000,- for equipment: 5 laptops for the researchers and PhD-students; and finally 100000,-x 3 for other operating expenses, such as expenses involving international conferences, arranging work-shop etc.

Implementation and follow up

At the end of the study a workshop will be organized for school nurses, doctors, teachers and researchers in the field. Apart from that, results of the study will be published in international journals as well as national journals of the professionals involved. If the proposed intervention proves to be effective, future studies can be set up for larger groups of adolescents.

Project Group and collaborating partners

The project group will consist of

- Sølvi Helseth, PhD, professor (project leader), Oslo University College, Faculty of Nursing
- Hilde Eide, PhD, Associate Professor, Oslo University College Faculty of Nursing
- Knut Løndal, MA, Associate professor, Oslo University College, Faculty of Education and International Studies
- Yngve Ommundsen, Professor, The Norwegian School of Sports Sciences
- Kirsti Riiser, PhD-student 1 (The RCT) Oslo University College, Faculty of Nursing and The Norwegian School of Sports Sciences
- *PhD student 2*
- *Research assistant.*
- *Technical support/cooperation*
- Lis Ribu, PhD, Associate Professor, Oslo University College, Faculty of Nursing.

At the Faculty of Nursing we have established a research group for lifestyle-interventions (GLIS) where we specifically focus on building competence on web-interventions (http://www.hio.no/content/view/full/42). Two large studies have already received financial support from the Norwegian Research Council (ie E:care, project leader Lis Ribu) and collaboration is established with NST (Norsk Senter for Telemedisin). The collaborating institutions/sections will be Faculty of Nursing, Faculty of Education and International Studies at Oslo University College, The Norwegian School of Sports Sciences and The Research Program at Oslo University College: Care, Health and Welfare which focuses on the development of knowledge and research within the health and social sectors. The leader of the Program is Professor Espen Dahl. Further, an informal network has been established between Oslo University College, the Child Growth Study at the Norwegian Public Health Institute, and the HEIA study at the University in Oslo with the purpose of providing each other with information and possible collaboration in future research on overweight and obesity during childhood. The project have been cooperating with "Stor&sterk" at Ullevål University Hospital in recruiting overweight adolescents for the two master thesis completed in the study (Berke 2009, Sanderud 2010, UMB). In addition we will work together with adolescents from "Stor &sterk" in the development of our web-solution.

Literature.

1. An JY, Hayman LL, Park YS, Dusaj TK, Ayres CG. Web-based weight management programs for children and adolescents: a systematic review of randomized controlled trial studies. ANS Advances in Nursing Science. 2009 Jul-Sep;32(3):222-40.
2. Arterburn DE. Obesity in children. BMJ Clin Evid. 2007:12:325. http://clinicalevidence.bmj.com/ceweb/conditions/chd/0325/0325_I1.jsp. Retrieved 040508.
3. Berenson GS, Srinivasan SR, Bao W, Newman WP 3rd, Tracy RE, Wattigney WA.Association between multiple cardiovascular risk factors and atherosclerosis in children and young adults. The Bogalusa Heart Study. N Engl J Med. 1998 Jun 4;338(23):1650-6.
4. Blair SN, Brodney S. Effects of physical inactivity and obesity on morbidity and mortality: current evidence and research issues. Med Sci Sports Exerc. 1999 Nov;31(11 Suppl):S646-62
5. Booth ML, Wilkenfeld RL, Pagnini DL, Booth SL, King LA. Perceptions of adolescents on overweight and obesity: the weight of opinion study. J Paediatr Child Health. 2008 May;44(5):248-52. Epub 2008 Jan 10.
6. Conolly MA, Johnson JA. Measuring quality of life in paediatric patients. *Pharmacoeconomics* 1999; 16:605-25
7. de Beer M, Hofsteenge GH, Koot HM, Hirasing RA, Delemarre-van de Waal HA, Gemke RJ.Health-related-quality-of-life in obese adolescents is decreased and inversely related to BMI.Acta Paediatr. 2007 May;96(5):710-4. Epub 2007 Mar 23
8. Eiser C., Morse R.. A review of measures of quality of life in children with chronic illness. *Arch Dis Child* 2001; 84:205-211
9. Epstein LH, Smith JA, Vara LS, Rodefer JS. Behavioral economic analysis of activity choice in obese children. Health Psychol. 1991. Vol. 10, pp. 311-316.

10. Fallon EM, Tanofsky-Kraff M, Norman AC, McDuffie JR, Taylor ED, Cohen ML, Young-Hyman D, Keil M, Kolotkin RL, Yanovski JA. Health-related quality of life in overweight and nonoverweight black and white adolescents. J Pediatr. 2005 Oct;147(4):443-50
11. Fitzmaurice G, Laird N, Ware J (2008). Applied longitudinal analysis. http://www.biostat.harvard.edu/~fitzmaur/ala/
12. Gerrish K Lacey A (2006) The research process in nursing. Blackwell Publishing Ltd.
13. Gillison FB, Standage M, Skevington SM. Relationships among adolescents' weight perceptions, exercise goals, exercise motivation, quality of life and leisure-time exercise behaviour: a self-determination theory approach. Health Education Research, *2006, 21*(6), pp. 836-847
14. Haraldstad K, Christophersen KA, Natvig GK, Eide H, Helseth S (2010). Health-related quality of life in children and adolescents: reliability and validity of the Norwegian version of the KIDSCREEN-52 questionnaire: a cross-sectional study. (Submitted)
15. Helseth S, Misvær N (2010). Adolescents' perceptions of quality of life: what it is and what matters. Journal of Clinical Nursing; 19-1454-1461
16. Hill AJ, Lissau I. Psychosocial factors. In: Burinat W, Cole T, Lissau I, Poskitt EME eds.Child and adolescent obesity: Causes and consequences, prevention and management. England: Camebridge University Press, 2002: 109-127
17. Hill, JO, Peters JC. Environmental contributions to the obesity epidemic. Science*,* 1998, Vol. 280. pp. 1371 – 1374
18. Hinkle E, Wiersma W and Jurs SG. Applied statistics for the behavioral sciences. Boston : Houghton Mifflin, 2003
19. Helseundersøkelsen i Nord-Trøndelag. Ung-HUNT 3. Spørreskjema for ungdom. 2006, http://www.hunt.ntnu.no/skjema%20hunt%203/ung%20hunt%203.pdf. Retrieved 050508
20. Kien CL, Chiodo AR. Physical Activity in Middle School–aged Children Participating in a School-Based Recreation Program. 2003, Arch Pediatr Adolesc Med. *Vol. 157:811-815.*
21. Kvale S, Brinkmann S (2009). Interviews : learning the craft of qualitative research interviewing. SAGE, LA, California.
22. Lien N, Kumar BN, Lien L. [Overweight among adolescents in Oslo]
23. Tidsskr Nor Laegeforen. 2007 Sep 6;127(17):2254-8. Norwegian.
24. Lissau I, Overpeck MD, Ruan WJ, Due P, Holstein BE, Hediger ML; Health Behaviour in School-aged Children Obesity Working Group. Body mass index and overweight in adolescents in 13 European countries, Israel, and the United States. Arch Pediatr Adolesc Med. 2004 Jan;158(1):27-33.
25. Leger LA, Mercier D, Gadoury C, Lambert J. The multistage 20 metre shuttle run test for aerobic fitness. 1988 J Sports Sci*, 6*, pp. 93-101
26. Markland, D. & Tobin, V. (2004). A modification of the Behavioral Regulation in Exercise Questionnaire to include an assessment of amotivation. *Journal of Sport and Exercise Psychology, 26,* 191-196
27. Molnár D, Livingstone B. Physical activity in relation to overweight and obesity in children and adolescents. Eur J Pediatr. 2000 Sep;159 Suppl 1:S45-55. Review.
28. Must A, Spadano J, Coakley EH, Field AE, Colditz G, Dietz WH The disease burden associated with overweight and obesity JAMA. 1999 Oct 27;282(16):1523-9.
29. Nowicka P, Flodmark CE. Barnövervikt i praktiken : evidensbaserad familjeviktskola. 2006. Lund : Studentlitteratur
30. McArdle WD, Katch FI, Katch VL. *Exercise Physiology,* fifth edition. 2001. Philadelphia Usa: Lippincott Williams & Wilkins
31. McArdle WD, Katch FI, Katch VL. Essentials of exercise physiology, second ed. 2000 Philadelphia : Lippincott Williams & Wilkins
32. Oude Luttikhuis H, Baur L, Jansen H, Shrewsbury VA, O'Malley C, Stolk RP, Summerbell CD. Interventions for treating obesity in children. *Cochrane Database of Systematic Reviews* 2009, Issue 1. Art. No.: CD001872. DOI: 10.1002/14651858.CD001872.pub2.
33. Pal DK. Quality of life assessment in children: a review of conceptual and methodological issues in multidimensional health status measures. J Epidemiol
34. Polit, D, Beck CT (2004) Nursing research: principles and methods Philadelphia, Pa. : Lippincott Williams & Wilkins
35. Raben A, Basse K, Rahbek AH et al. BMI prevalence underestimated by 50% by using self-reported data on weight and height. Int J Obes Realt Metab Disord 2002;23(suppl1), 12.
36. Rangul V, Holmen TL, Midthjell K, Kurtze N. Reliabilitet og validitet på tre spørreskjemaer om fysisk aktivitet blant ungdom. Hva måler de egentlig. 2006, Norsk Epidemiologi, 16 (supplement 1), p. 22.
37. Ravens-Sieberer U, Redegeld M, Bullinger M. Quality of life after in-patient rehabilitation in children with obesity. Int J Obes Relat Metab Disord. 2001 May;25 Suppl 1:S63-5.

38. Rising R, Harper IT, Fontvielle AM, Ferraro RT, Spraul M, Ravussin E. (1994). Determinants of total daily energy expenditure: Variability in physical activity. *American Journal of Clinical Nutrition*, Vol. 59: 800-804
39. Sallis JF, Simmons-Morton BG, Stone EJ, et al. Determinants of physical activity and interventions in youth. 1992 Med Sci Sports Exerc. Vol. 24(6 suppl), pp. 248-257
40. Schwarzer, R. (1993) Measurement of perceived self-efficacy. Psychometric scales for cross-cultural research. Berlin, Germany: Freie Universität Berlin, Inst. Fur Psychologie
41. SEF. Vekt - helse. Rapport nr 1/2000. Oslo: Statens råd for ernæring og fysisk aktivitet.
42. Singh AS, Mulder C, Twisk JW, van Mechelen W, Chinapaw MJ. Tracking of childhood overweight into adulthood: a systematic review of the literature. Obes Rev. 2008 Mar 5. [Epub ahead of print]
43. Spilker, B. (Ed). Quality of life asessments in clinical trials. 1990, Raven Press, New York
44. Stone EJ, McKenzie TL, Welk GJ and Booth ML (1998). Effects of physical activity interventions in youth: review and synthesis. 1998 American Journal of Preventive Medicine, Vol. 15, No. 4, pp. 298-315
45. Summerbell CD, Waters E, Edmunds LD, Kelly S, Brown T, Campbell KJ. Interventions for preventing obesity in children. Cochrane Database of Systematic Reviews 2005, Issue 3. Art. No.: CD001871. DOI: 10.1002/14651858.CD001871.pub2
46. Summerbell CD, Ashton V, Campbell KJ, Edmunds L, Kelly S, Waters E. Interventions for treating obesity in children. Cochrane Database of Systematic Reviews 2003, Issue 3. Art. No.: CD001872. DOI: 10.1002/14651858.CD001872.
47. Summerbell CD, Waters E, Edmunds L, Kelly SAM, Brown T, Campbell KJ. Interventions for preventing obesity in children. *Cochrane Database of Systematic Reviews* 2005, Issue 3. Art. No.: CD001871. DOI: 10.1002/14651858.CD001871.pub2.
48. Vilimas K, Glavin K, Donovan ML. [Overweight among eight and twelve-year-old children in Oslo in 2004] Tidsskr Nor Laegeforen. 2005 Nov 17;125(22):3088-9. Norwegian
49. Whitaker RC, Wright JA, Pepe MS, Seidel KD, Dietz WH. Predicting obesity in young adulthood from childhood and parental obesity. N Engl J Med. 1997 Sep 25;337(13):869-73.
50. World Health Organisation (WHO). Obesity and overweight. Fact sheet N°311. September 2006. http://www.who.int/mediacentre/factsheets/fs311/en/print.html. Retrieved 040508.

.
